# Capture of *Escherichia coli* O157:H7 Using Immunomagnetic Beads of Different Size and Antibody Conjugating Chemistry

**DOI:** 10.3390/s90200717

**Published:** 2009-01-29

**Authors:** Shu-I Tu, Sue Reed, Andrew Gehring, Yiping He, George Paoli

**Affiliations:** United States Department of Agriculture, Agricultural Research Service, Eastern Regional Research Center, 600 East Mermaid Lane, Wyndmoor, PA 19038, USA

**Keywords:** *Escherichia coli*, immunomagnetic beads, time-resolved fluorescence, antibody linkage

## Abstract

Immunomagnetic beads (IMB) were synthesized using anti-*Escherichia coli* O157 antibodies and magnetic beads of two different sizes (1 μm and 2.6 to 2.8 μm) that contained a streptavidin coating, activated carboxyl groups or tosylated surfaces. The synthesized IMB, together with a commercially available IMB, were used to capture different strains of *E. coli* O157:H7 and *E. coli* O157:NM. The *E. coli* capture was measured by the time resolved fluorescence (TRF) intensity using a sandwich assay which we have previously demonstrated of having a sensitivity of 1 CFU/g after 4.5 hour enrichment [1]. The analyses of measured TRF intensity and determined antibody surface concentration indicated that larger beads provided higher response signals than smaller beads and were more effective in capturing the target of interest in pure culture and ground beef. In addition, while each type of IMB showed different favorable capture of *E. coli* O157:H7, streptavidin-coated IMB elicited the highest response, on average. Streptavidin-coated IMB also provided an economic benefit, costing less than $0.50 per assay. The results could be used to guide the proper choice of IMB for applications in developing detection processes for *E. coli* O157:H7.

## 1. Introduction

Due to recent high profile outbreaks of *E. coli* O157:H7 in spinach [2] and *Salmonella* Saintpaul in raw produce [3], the safety of our food has been of highest concern. From the farm where the food is produced, to the handling practices of manufacturers, to our own kitchens, food safety involves all stages of food production and consumption. Therefore, the need not only for the eradication of food-borne pathogens exists, but also their rapid and sensitive detection once they enter the food chain.

Traditional methods of detecting food-borne pathogens include enrichment, plating to selective and/or differential agar, and biochemical/serological confirmation [4,5]. This process can take days and is labor intensive. Immunomagnetic separation (IMS) offers advantages over traditional pathogen enrichment processes. Superparamagnetic particles are coated with antibodies against the target of interest, forming immunomagnetic beads (IMB). The specificity of the antibody coupled with the magnetic properties of the bead, allows a target organism to be separated from a food matrix and background microflora, and concentrated into a smaller sample volume [6].

IMS also has the advantage of being very versatile. Many methods of detection can be used to quantify and/or identify the captured bacteria. IMB can be plated directly onto a selective medium [7] or used as a solid support for an ELISA [8]. In addition, various types of IMB have been used to capture and concentrate target pathogen cells, which are then eluted from the beads and subjected to PCR identification [9-11]. Another common method of detection involves adding a secondary antibody to create a sandwich-immunoassay. Variations on this assay are almost limitless. Secondary antibodies labeled with alkaline phosphatase [12], horseradish peroxidase [13], and fluorescein isothiocyanate (FITC) [14] have been utilized with promising results.

In recent years, time-resolved fluorescence (TRF) has also been used quite extensively as a means of pathogen detection. TRF utilizes lanthanide chelate labels which produce intense fluorescent signals with a long half-life (10^-3^ to 10^-6^ s). This long decay time coupled with a large Stokes' shift (>200 nm) and narrow emission peak allow the chelate label to be read after nonspecific background has already decayed. This allows for increased sensitivity and enhanced signal-to-noise ratio. IMS combined with TRF has been successful in the detection of 10 cfu/mL of *E. coli* O157:H7 in apple cider [15], 1 cfu/g of *E. coli* O157:H7 in ground beef [1], and 4 cfu/g of both *Salmonella* and *E. coli* O157 from germinated alfalfa sprouts [16].

In this study, we have chosen a combination of IMS and TRF as a sensitive process to detect different strains of *E. coli* O157. The study mainly focused on the detection efficiency of using IMB of various sizes which consisted of different types of chemical linkage for conjugating the capture antibody to the superparamagnetic particle. Two types of covalent coupling, Schiff-base and tosylation, along with biotin-streptavidin interaction were used to attach anti-*E. coli* O157:H7 antibodies to different beads. In addition, we have also examined the efficiency of beads with different size but of a similar density in capturing the targeted pathogens. In conjunction with the efficiency study, a cost analysis was also conducted to reveal the economics of using different IMB. The result could be used as a guide for designing the proper choice of IMB to capture *E. coli* O157:H7 for TRF measurement.

## 2. Methods and Materials

### 2.1. Immunomagnetic beads

Two sizes of IMB consisting of anti-*E. coli* O157 antibodies attached via different linking chemistries were examined for the capture and detection of *E. coli* O157:H7 (Table 1). IMB from a commercially available stock were also examined. Small beads with 1 μm diameters include P(S/V-COOH) Mag/Encapsulated (IMB-C1; Bangs, Fishers, IN), and Dynabeads MyOne Streptavidin C1 (IMB-S1; Invitrogen Dynal AS, Oslo, Norway) and Dynabeads MyOne Tosylactivated (IMB-T1; Invitrogen). Larger beads having diameters between 2.6 and 2.8 μm include COMPEL™ Magnetic, COOH-modified microspheres (IMB-C; Bangs), and Dynabeads M-280 streptavidin (IMB-S; Invitrogen) and Dynabeads M-280 tosylactivated (IMB-T; Invitrogen). Commercially available Dynabeads anti-*E. coli* O157 (IMB-D; Invitrogen) were also used for comparison. Information about these beads is proprietary. However, the size and density were estimated by microscopic examination and discontinuous sucrose density gradient centrifugation, respectively [17]. In addition, the stock concentration of beads/mL was estimated by manual counting with a Petroff-Hausser counting chamber (Hausser Scientific, Horsham, PA) as described below in Section 2.4.

### 2.2. Conjugation of antibodies to IMB

Both sizes of carboxylated beads were conjugated with 16 μg/mg of goat anti-*E. coli* O157:H7 antibodies (KPL, Gaithersburg, MD) using a PolyLink Protein Coupling Kit (Bangs) following the manufacturer's instructions. Water soluble carbodiimide was used to activate the carboxyl groups on the surface of carboxylated beads. This reaction creates an active ester, which binds to the primary amine groups of the antibody. Biotinylated antibodies were conjugated onto beads containing a layer of streptavidin covalently attached to the bead surface. The affinity of streptavidin for biotin represents a strong noncovalent interaction, having a dissociation constant of ∼10^-15^ M, rivaling that of covalent bonds. Biotinylated goat anti-*E. coli* O157:H7 antibodies (KPL) were conjugated to both IMB-S and IMB-S1 at concentrations of 6 μg/mg and 20 μg/mg, respectively, as follows: suspensions of 100 μL of beads were mixed with 900 μL phosphate buffered saline (PBS; Sigma Chemical, St. Louis, MO). Six or twenty microliters of biotinylated goat anti-*E. coli* O157:H7 antibody (KPL, 1 mg/mL stock concentration) were added and allowed to bind at room temperature for 30 minutes with gentle rocking on a Specimix (Barnstead International, Dubuque, IA). After conjugation, the beads were washed 4 times with a solution of 20 mM PBS, 150 mM NaCl, 2 mM EDTA, and 0.5% BSA. After the final washing, the beads were resuspended in 1 mL of the same solution and stored at 2–8°C. Goat anti-*E. coli* O157:H7 antibody (KPL) was conjugated to IMB-T and IMB-T1 at concentrations of 20 μg/mg and 40 μg/mg, respectively, following the manufacturer's guidelines. Hydroxy groups on tosylactivated beads were treated with p-toluensulphonyl chloride, resulting in a sulphonyl ester which binds to amino or sulfhydryl groups of the antibody. The optional addition of 0.1% BSA after the first 10 minutes of conjugation was applied. The amount of conjugated antibody and linking chemistry of the IMB-D are considered proprietary by the manufacturer.

### 2.3. Bound protein determination of IMB

The amount of antibody bound to the IMB was determined by absorbance at 280 nm using a Cary 50 spectrometer (Varian, Inc., Palo Alto, CA). Standard curves of absorbance vs. concentration for each type of antibody were generated by measuring the absorbance of various antibody concentrations at 280 nm using a quartz cuvette. The extinction coefficients at 280 nm derived from the standard curves were used for estimating free antibody concentrations after conjugation. The amounts of antibody bound to beads were calculated from the differences between total-applied prior to and free-remained after the conjugation. The calculated concentrations of antibody bound to the beads are shown in Table 2.

### 2.4. Selection of E. coli strains and enumeration

*E. coli* O157:H7 strains B1409 (human stool sample; Centers for Disease Control and Prevention, Atlanta, GA), SEA 13B 88 (apple cider outbreak; Food and Drug Administration, Rockville, MD), and 380-94 (salami outbreak; Food Safety and Inspection Service, Washington, DC) along with *E. coli* O157:NM strain MF 13180-NM (FSIS) and non-O157 strain K12 (source unknown) were grown overnight in 25 mL mEC broth (Becton, Dickinson, and Company, Sparks, MD) at 37°C with shaking at 160 rpm (New Brunswick Scientific, Edison, NJ). After overnight enrichment, bacteria in 1 mL of each culture were pelleted by centrifugation (Eppendorf, Westbury, NY) and resuspended in PBS. The bacteria were then diluted 1:100 in PBS and enumerated using a Petroff-Hausser counting chamber (Hausser Scientific). Six microliters of the 1:100 diluted culture were placed onto the counting chamber slide. The slide consists of 25 0.2 mm×0.2 mm squares. The bacteria in five random squares were counted in duplicate, and cell concentration in cells/mL calculated. Serial dilutions of each *E. coli* strain were prepared in PBS from 1×10^8^ to 1×10^2^ cells/mL following enumeration. Two hundred microliters of the diluted suspensions were subjected to the TRF immunoassay as described in section 2.6.

### 2.5. Preparation of cell suspension and inoculation of ground beef

For the ground beef experiments, a suspension of 1 mL PBS containing 25 cells was prepared and used to inoculate 25 g of ground beef in a stomacher bag with mesh filter (Fisher Scientific, Pittsburgh, PA). The inoculum was manually massaged into the ground beef followed by the addition of 225 mL of mEC broth. The sample was then mixed in a stomacher (Seward Medical Limited, London, UK) on low for 30 s, followed by enrichment for 24 h at 37°C with shaking at 160 rpm. After enrichment, aliquots of 200 μL from the side of the mesh filter without ground beef were withdrawn for IMS capture and TRF assay as described in section 2.6. A second aliquot of ground beef was inoculated with 1mL PBS to serve as a blank. The blank was run alongside the sample to gauge the growth of background organisms. The post-enriched sample was also plated onto Sorbitol MacConkey agar supplemented with cefixime and tellurite (CT-SMAC; Becton, Dickinson, and Company) to enumerate the growth of O157:H7 in ground beef. The post-enrichment blank was plated on both plate count agar (PCA; Becton, Dickinson, and Company) and CT-SMAC to enumerate background organisms and ensure no indigenous *E. coli* O157:H7 in the ground beef.

### 2.6. Immunomagnetic separation and time-resolved fluorescence detection

Immunomagnetic separation (IMS) was carried out using the KingFisher magnetic particle processor (Thermo Fisher, Waltham, MA). The KingFisher automatically transfers beads between binding and washing steps. The entire assay was performed in black 96-well microtiter plates (Nalge Nunc, Rochester, NY). To row A, suspensions of cultured *E. coli* cells or enriched ground beef samples with a volume of 200 μL were added to 20 μL of beads. To rows B and D, 200 μL of washing buffer diluted 1:25 from wash concentrate (Wallac Oy, Turku, Finland) supplemented with 0.5% Tween 20 (Acros Organics, Fairlawn, NJ) was added. Goat anti-*E. coli* O157:H7 (KPL) was labeled with europium using a DELFIA^®^ Eu-Labeling Kit (Perkin Elmer LAS, Boston, MA) following the manufacturer's specifications. The labeled stock was stored frozen at -20°C. Prior to the assay, the europium-labeled detection antibody was diluted in assay buffer (Wallac Oy) supplemented with 0.1% Tween 20 to a concentration of 1 μg/mL, filtered through a 0.45 μm syringe filter (Nalge Nunc), and 200 μL added to each well of row C. Lastly, 200 μL of enhancement solution (Wallac Oy) was added to row E. The samples were processed in the KingFisher, which magnetically transfers the beads from row to row. After initial binding of bacteria to beads in row A for 15 min, the bead/bacteria complexes were washed for 1 min in row B. The washed complexes were then transferred to row C and allowed to bind with the europium-labeled detection antibody for 1 h. After another 1 min wash in row D, the complexes were transferred into the enhancement solution in row E. Proprietary chelators in the enhancement solution extracted the europium to form an Eu-chelator that emitted strong fluorescent signal at 615 nm. The fluorescence intensity displayed as counts per second (CPS) was measured using a VICTOR^2^ 1420 multilabel counter (Perkin-Elmer Wallac, Waltham, MA).

### 2.7. Data analysis

Data were replicated at least three times, with averages and percent error reported. The percent error was calculated by dividing the standard deviation among replicates by the average signal obtained by the TRF assay and multiplying by 100. Microsoft Excel was applied to determine the statistical parameters of variance and standard deviation using Microsoft Excel spreadsheet. Normalized responses per number of applied beads data were calculated by dividing the TRF response by the number of beads used in the assay for further evaluation on the interaction of the bacteria with the beads.

### 2.8. IMB cost analysis

The cost of each IMB per assay was estimated by taking into consideration the cost of the beads, antibody, and coupling kit (for carboxylated beads only) used in the conjugation of each IMB. For example, IMB-S costs $899/10mL. Biotinylated goat anti-*E. coli* antibody costs $400/1 mL. In the labeling procedure, 100 μL of IMB are labeled with 6 μL antibody. Therefore, the beads are $8.99 for 100 μL, and the antibody is $0.40 per μL or $2.40 per 6 μL. Adding the cost of the beads and antibody together, the total cost for preparing 1mL of IMB-S is $11.39. Since 20 μL of beads are used per assay, the cost per assay is $0.23.

## 3. Results and Discussion

### 3.1. Optimization of beads

Table 1 displays the properties of each IMB, including size, density, stock concentration (number of prepared beads/mL), and beads/assay (number of beads in 20 μL of the stock). Table 2 shows the amount of antibody applied in the binding process (based on manufacturer's suggestions and specifications), the concentration of antibody bound to the beads, and the surface antibody concentration per total surface area (SA). The smaller beads contained a higher concentration of antibodies bound to their surface than the larger beads, with the exception of the carboxylated beads. The total surface area for the 1 μm beads was greater than that of the larger beads, thereby providing more sites to which the antibodies could bind. Concerning the carboxylated beads, the same amount of antibody was applied to both sizes during the binding process. With the same amount of antibody to cover a greater surface area, it stands to reason that the surface antibody concentration would be less for the 1 μm beads compared to the 2.6 μm beads.

Additionally, a cost analysis was done to give an approximate cost of each IMB per assay (20 μL), taking into account the amount of antibody applied, amount of beads used in the labeling process, and coupling kits where applicable. Common reagents/solutions were not included in the cost analysis since they were made in-house from lab grade chemicals. Cost analysis results are also shown in Table 1. Both sizes of carboxylated beads had the lowest cost per assay volume, while the IMB-D appeared to be more expensive.

### 3.2. Capture of E. coli O157:H7 in suspension

**Figure 1.**
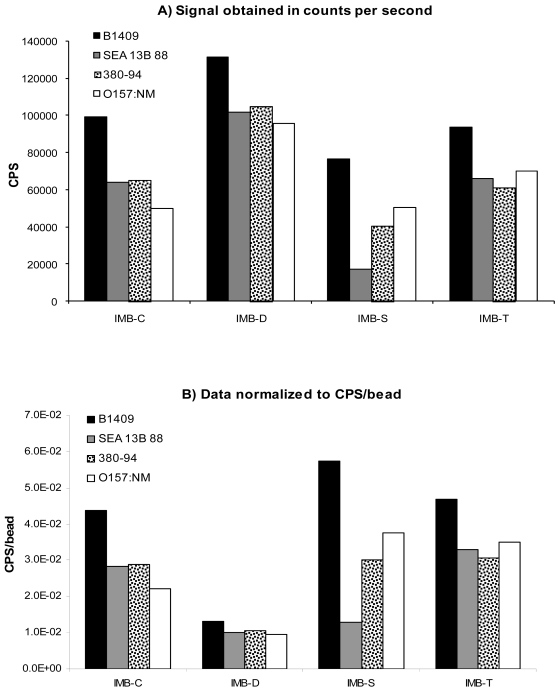
Response obtained using 2.6–2.8 μm IMB in the capture of 1×10^7^ cells/mL of *E. coli* O157:H7 and *E. coli* O157:NM from suspension. A) shows the data obtained in counts per second (CPS) while B) shows the results normalized to the number of beads used per assay. The average percent error among replicates was ±7.8%.

*E. coli* O157:H7 strains B1409, 380-94, and SEA 13B 88 along with *E. coli* O157:NM were diluted in PBS and assayed with each IMB. *E. coli* K12 was also tested with each IMB for cross-reactivity. No detectable signal was observed for K12 (data not shown). Figure 1 shows the results obtained using the 2.6–2.8 μm beads to capture 1×10^7^ cells/mL of *E. coli* O157. The top graph, A, shows the capture of bacteria as measured by the fluorescence intensity (CPS) for each strain tested with each IMB. For all strains, IMB-D provided the highest response, followed by IMB-C or IMB-T. IMB-S provided the lowest response for all strains except O157:NM. The data in the bottom graph, B, has been normalized to the number of beads contained in the 20 μL assay volume (as shown in Table 1). This normalization reflects the signal per bead, allowing all IMB to be directly compared to one another and thus, eliminated the complications of having different number of beads used in the assay. When comparing the normalized data obtained for the 2.6–2.8 μm IMB, the IMB-S provided the highest response, while that associated with IMB-D was the lowest. This is the exact opposite trend as seen in the top graph containing the CPS results. This is due to the fact that more beads were present in 20 μL of IMB-D than the others. Therefore, with similar CPS observed, the IMB type containing the fewer number of beads per 20 μL provides a higher normalized signal. Such is the case with the IMB-S. There are about twice the number of beads in 20 μL of IMB-C and IMB-T than in the same volume of IMB-S. IMB-D on the other hand, contains almost 10 times the number of beads in 20 μL as does IMB-S.

**Figure 2.**
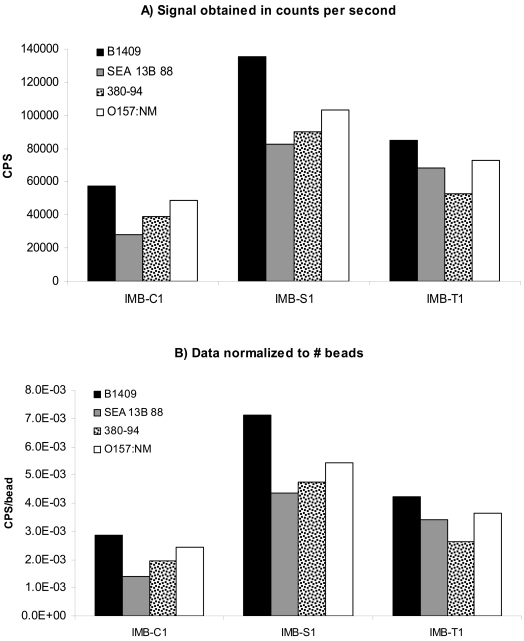
Response obtained using 1 μm IMB in the capture of 1×10^7^ cells/mL of *E. coli* O157:H7 and *E. coli* O157:NM from suspension. A) shows the data obtained in counts per second (CPS) while B) shows the results normalized to the number of beads used per assay. The average percent error among replicates was ±8.7%.

In addition to the beads used above, 1 μm carboxylated, streptavidin-coated, and tosylactivated IMB were also examined. Under the same experimental conditions and using the same concentration of *E. coli*, the results in Figure 2 were obtained.

These results are a little different than those obtained from using the larger beads. First, the data expressed in CPS and the normalized data show similar trends. For both A and B, IMB-S1 provided the highest response, followed by IMB-T1 and IMB-C1. This is due to the fact that the number of beads per 20 μL was just about the same for all three 1 μm IMB. In 20 μL assay volumes, there were 2×10^7^ beads for both IMB-C1 and IMB-T1, and an average of 1.9×10^7^ beads for IMB-S1. Because the number of beads in 20 μL of each IMB was virtually the same, the trends observed in the CPS data mirrors that of the normalized data. Secondly, the normalized signals generated by IMB-S1 and IMB-T1 are not as intense as those generated by their larger counterparts, even though the antibody surface concentration of the 1 μm beads is greater than that of the larger IMB. In order to explore this observation further, a set of experiments using the same number of beads/20 μL assay volume for both sizes and all types of IMB was undertaken, in order to get a more direct comparison. This would eliminate any effects by the use of differing number of beads/assay for each IMB. For the carboxylated beads, the lower signal generated by IMB-C1 could be due to less antibody surface or it could be due to the size, as was explored further.

### 3.3. Effects of bead size

**Figure 3.**
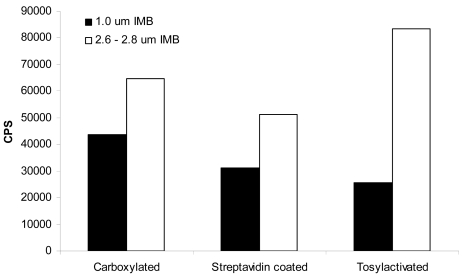
Comparison of IMB using 1×10^6^ beads/20 μL assay volume. Each IMB was diluted in PBS to contain 1×10^6^ beads/20 μL and tested for capture with 1×10^7^ cells/mL *E. coli* O157:H7 strain B1409. The larger sized beads provided greater capture of bacteria than those of a smaller size. The average percent error among three independent experiments run in triplicate was ±23%.

We have studied the hydrodynamic factors associated with beads of different sizes and densities in previous reports [18, 19]. Our findings revealed that larger beads were more effective in capturing bacteria than smaller beads of the same density. This is due to the fact that larger beads can travel through a larger volume of sample solution and thus, have more opportunity to interact with the target of interest. The same effects were seen with carboxylated, streptavidin-coated, and tosylactivated beads of different sizes. By comparing part B of both Figures 1 and 2, it is clear that for each IMB type, the larger 2.6–2.8 μm beads elicited greater normalized signals than the same type of the 1 μm size. However, it is hard to compare these two figures with out the bias of the number of beads used per assay. In order to get a better comparison of the effect of the bead size on the capture of *E. coli* O157:H7, each IMB was diluted in PBS to contain 1×10^6^ beads/20 μL assay volume. The IMB-D was not used in this experiment because it was only commercially available in the 2.8 μm size at the time of this study. Figure 3 shows the results obtained when a consistent number of beads were used.

### 3.4. Trial in the ground beef system

**Figure 4.**
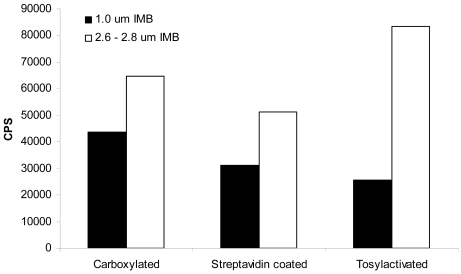
Capture of *E. coli* O157:H7 strain B1409 from ground beef. Strain B1409 was inoculated into ground beef at a concentration of 1 cfu/g, enriched for 24 h in mEC broth, and assayed with each IMB. A) shows the results obtained in counts per second, with all IMB producing a similar response (*P* = 0.64). However, when the data is normalized to the number of beads used per assay (B), the results were quite different with IMB-C, IMB-S, and IMB-T giving the greater signal. The data obtained is from three independent experiments run in duplicate. The average percent error among these replicates was ±22%.

After a 24 h enrichment in mEC broth, both the blank and sample were assayed with each type of IMB. The results are found in Figure 4.

The total aerobic plate count for background microflora revealed 2.0×10^9^ cfu/mL of background organisms, no *E. coli* O157:H7, and the CT-SMAC plating revealed 1.4×10^9^ cfu/mL of *E. coli* O157:H7 in the ground beef sample inoculated with 1 cfu/g. The response data in Figure 4 shows all IMB to generate similar signals in the TRF assay. In fact, an analysis of variance shows all beads to be statistically similar (*P* = 0.64). However, when the data is normalized to the number of beads used in the assay, it becomes clear that three IMB have advantages over the others. IMB-S, IMB-C, and IMB-T all elicit higher signals per bead than the others. This is consistent with the observations in suspension that the larger beads capture more bacteria and thus elicit a higher signal. In addition, the normalized data is also consistent with results of IMB-D as seen in suspension. Therefore, it can be said with confidence that neither background flora present in ground beef nor the matrix of ground beef itself interferes with the capture and detection of *E. coli* O157:H7.

## 4. Conclusions

We have shown in this report and others that larger size IMB are more effective in the capture of a target of interest. While the surface antibody concentration of IMB-S and IMB-T was less than that of the smaller beads, they still elicited a higher response. This is due to the ability of the larger beads to interact with target organism in larger volumes as proposed in our previous study [18, 19]. IMB-C, on the other hand, generated higher signals than IMB-C1. However, IMB-C also had a higher concentration of antibodies bound to their surface. Therefore, it cannot be exclusively concluded that the size of the carboxylated beads was the only factor in their generation of higher signals than the IMB-C1. In this report, we have also expanded on this idea to include IMB utilizing three different surface chemistries used to link the capture antibody to the magnetic particles. Different strains of *E. coli* O157 react differently to the same beads. This could be due to antigenic differences between the strains. For all strains tested with the 1 μm beads, the streptavidin-coated beads elicited the highest response. For the larger beads, IMB-S also provided the highest normalized signal for strains B1409 and O157:NM. All IMB except IMB-D produced similar signals with strain 380-94. Strain SEA 13B 88 was the only strain which saw IMB-S produce the lowest signal. Again, different antigenic features of this strain are the most probable explanation as to the lower capture with the IMB-S. While each type of antibody linkage tested favorably, especially in a real world sample, the streptavidin-coated beads may hold a slight advantage. On the whole, streptavidin-coated particles performed very well in the capture of *E. coli* O157:H7 and O157:NM. Perhaps the orientation of the antibodies on the surface of the streptavidin-coated beads allows for enhanced capture. In addition, streptavidin-coated IMB also provide an economic benefit, costing under $0.50 per assay.

## Figures and Tables

**Table 1.** IMB properties.

**Bead**	**Size** **(μm)**	**Density** **(g/mL)**	**Antibody linking chemistry**	**Stock conc.** **(beads/mL)**	**Beads per assay***^a^*	**Cost per assay***^a^*
IMB-C	2.6	1.2	Amide bond*^b^*	1.13E+08	2.26E+06	$0.14
IMB-C1	0.96	1.347	Amide bond*^b^*	1.0E+09	2.0E+07	$0.19
IMB-D	2.8*^c^*	1.3*^d^*	Proprietary	5.0E+08*^e^*	1.0E+07	$2.03
IMB-S	2.8	1.4	Biotin-streptavidin interaction*^f^*	6.7E+07	1.34E+06	$0.23
IMB-S1	1.05	1.8	Biotin-streptavidin interaction*^f^*	9.5E+08*^g^*	1.9E+07	$0.49
IMB-T	2.8	1.4	Amine bond*^h^*	1.0E+08	2.0E+06	$0.28
IMB-T1	1.08	1.7	Amine bond*^h^*	1.0E+09	2.0E+07	$0.28

a 20 μL of IMB used per assay.

b Carbodiimide activated carboxyl groups on beads bind with primary amines on antibodies through amide bonds.

cd Size and density estimated by microscopic examination and sucrose density gradient centrifugation, respectively.

e Enumeration of beads/mL was estimated using Petroff-Hausser counting chamber. The # beads/assay was calculated using this value.

f Biotinylated antibody binds to streptavidin-coated surface of beads through biotin-streptavidin interaction.

g Median value of beads estimated by manufacturer to be 7–12×10^8^ beads/mL.

h *p*-toluensulphonyl chloride activated hydroxy groups on beads bind with amino groups on antibodies through amine bonds.

**Table 2.** Bound antibody concentrations.

**Bead**	**Applied** **(μg/mg)**	**Bound** **(μg/mg)**	**% bound**	**Beads/mg**	**Total SA** **(μm^2^)**	**Surface antibody concentration** **(fg***^a^***/μm^2^)**
IMB-C	16	15.3	96	9.0E+07	1.9E+09	8.1
IMB-C1	16	12.2	76	1.6E+09	4.6E+09	2.7
IMB-S	6	0.78	13	6.7E+07	1.7E+09	0.5
IMB-S1	20	3.1	16	9.5E+08	3.3E+09	0.9
IMB-T	20	9.6	48	6.7E+07	1.7E+09	5.6
IMB-T1	40	38.4	96	1.0E+09	3.7E+09	10.4

a femtogram (10^-15^ g)
